# Genomic Divergence Between *Octopus vulgaris* and Its Undescribed Sister Species From the South Atlantic and Indian Ocean

**DOI:** 10.1002/ece3.73235

**Published:** 2026-03-16

**Authors:** Arsalan Emami‐Khoyi, Gareth N. Fee, Jannes Landschoff, Michael D. Amor, Charles Griffiths, Yves Cherel, Peter R. Teske

**Affiliations:** ^1^ Institute of Wildlife Management and Nature Conservation Hungarian University of Agriculture and Life Sciences Gödöllő Hungary; ^2^ Centre for Ecological Genomics and Wildlife Conservation, Department of Zoology University of Johannesburg Auckland Park South Africa; ^3^ Department of Biological Sciences University of Cape Town Cape Town South Africa; ^4^ Sea Change Project, Sea Change Trust Cape Town South Africa; ^5^ Department of Aquatic Zoology Western Australian Museum Welshpool Western Australian Australia; ^6^ Centre D'etudes Biologiques de Chizé (CEBC), UMR 7372 du CNRS‐La Rochelle Université Villiers‐en‐Bois France

**Keywords:** Cephalopoda, cryptic species, genome, mitogenome, *Octopus vulgaris* complex, phylogenomic analysis

## Abstract

Molecular data are widely used to resolve complex phylogenetic relationships between cryptic species, particularly in cases where morphological features are insufficient to confirm taxonomic distinctness. For benthic shallow‐water octopuses, several successes and failures have been reported when attempting to delineate species using individual nuclear or mitochondrial markers. In this study, we investigated the potential of shallow random shotgun sequencing to assess the phylogenetic placement of an undescribed southern hemisphere lineage within the *Octopus vulgaris* species complex, which could not be conclusively delimited using single‐marker approaches. A total of 338 nuclear loci, along with complete mitochondrial genomes, were generated for two specimens presently classified as *Octopus vulgaris* (Type III) that originated from the southeastern Atlantic coast of South Africa and Amsterdam Island in the southern Indian Ocean. Our combined phylogenomic approach reveals that this lineage is genetically distinct from *O. vulgaris* sensu stricto (ss) from the Mediterranean and the northeast Atlantic, as well as from the closely related *O. sinensis* from East Asia. A further separation of *O. vulgaris* (Type III) into distinct South African and Amsterdam Island lineages cannot be proven. These findings add to the growing body of evidence that supports *O. vulgaris* Type III as a genetically distinct lineage within the *O. vulgaris* species complex, and emphasise that the taxonomic classification of this southern hemisphere lineage warrants re‐evaluation.

## Graphical abstract

*Octopus vulgaris* Type III — an undescribed Southern Hemisphere lineage occurring along the South African coast and Amsterdam Island — is genomically distinct from *O. vulgaris* sensu stricto and *O. sinensis*, as inferred from phylogenetic analyses of 338 nuclear loci and complete mitochondrial genomes, supporting the need for taxonomic re‐evaluation of this species complex.

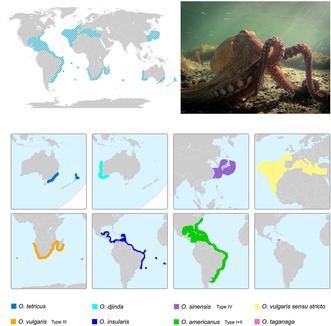

## 1 Introduction

Cryptic species are ubiquitous among marine invertebrates (Knowlton 1993). Within the Cephalopoda, crypsis is particularly common among benthic octopuses, as they often lack clearly delimiting, consistent morphological traits (Norman et al. 2013). The genus *Octopus* Cuvier, 1797 has long been considered a ‘catch‐all’ genus due to the subtle character differences among its species.

Molecular investigations, however, have revealed that the genus is polyphyletic, indicating the necessity for substantial taxonomic revision. The type species of the genus, *Octopus vulgaris* Cuvier, 1797, which is also the most extensively studied octopus worldwide, is now widely recognised as a species complex comprising multiple geographically isolated taxa (Kaneko et al. 2011; Norman et al. 2013; Sales et al. 2013; Amor et al. 2014; de Luca et al. 2014; Amor, Laptikhovsky, et al. 2017; Amor, Norman, et al. 2017; Avendaño et al. 2020; Borges et al. 2022; Fee et al. 2024).

*Octopus vulgaris* was originally described from the Mediterranean Sea and, based on morphological similarities with octopuses elsewhere, has subsequently been documented in temperate and tropical waters worldwide (Warnke et al. 2004; Guerra et al. 2010). Initial genetic studies, based on a small number of mitochondrial markers such as *16S rRNA* and *cox3*, supported the hypothesis of a monophyletic cosmopolitan species, as populations from the Mediterranean, South Africa, Brazil, Senegal, Taiwan, Tristan da Cunha, and Venezuela formed a single clade (Oosthuizen et al. 2004; Warnke et al. 2004). However, more recent studies using a wider range of nuclear and mitochondrial markers have supported the hypothesis of several geographically distinct species (Amor et al. 2014; Amor, Laptikhovsky, et al. 2017; Amor, Norman, et al. 2017; Amor et al. 2019).

**FIGURE 1:**
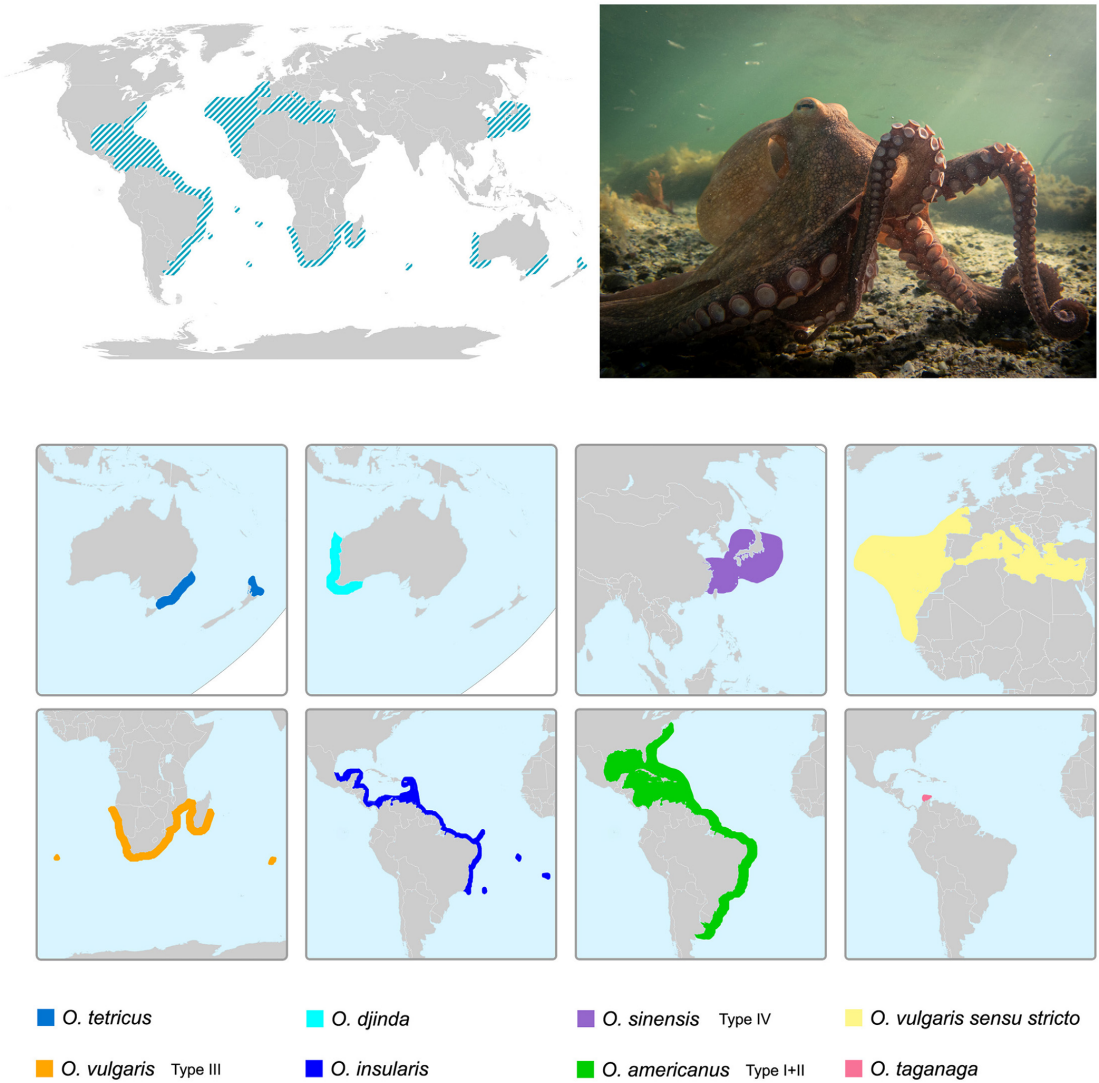
Distribution of *Octopus vulgaris* species complex across the globe (top), and the delineation of new species in the complex with their approximate distribution areas (bottom). The figure is based on Amor et al. (2019) and Avendaño et al. (2020).

Subsequent to these studies, several new species have been described within the *O. vulgaris* species complex: *Octopus americanus* Froriep, 1806 was determined to be the valid species name for the octopus common to the Atlantic coasts of the Americas (Avendaño et al. 2020), and *O. sinensis* A. d'Orbigny, 1834 was validated as the name for the East Asian common octopus (Gleadall 2016). Furthermore, contrary to the hypothesis that *O. vulgaris* has a circumglobal distribution, it was found that relatively small geographic distances can act as barriers to gene flow, leading to speciation within this group (Amor et al. 2024) (Figure 1).

As early as 1998, field observations, combined with morphological analyses of museum specimens, indicated that the South African population of *O. vulgaris* could represent a distinct species (Roeleveld 1998). More recent studies have supported this idea, and it was hypothesised that individuals from Tristan da Cunha Island in the South Atlantic, across the temperate southern African coastline of Namibia and South Africa, to Amsterdam Island in the Indian Ocean, belong to a distinct species within the *O. vulgaris* species complex (Norman et al. 2013; Amor, Laptikhovsky, et al. 2017). Of the six hypothesised species ‘Types’ within the *O. vulgaris* species complex reported by Norman et al. (2013), the widespread southern hemisphere *O. vulgaris* Type III is the least studied, and the only taxon that has not yet been redescribed.

In species delimitation using molecular approaches, it is not uncommon for a single genetic marker to be insufficient for resolving taxonomic uncertainties, since such markers only capture a limited portion of the genomic variation that encompasses the species' full evolutionary history (DeFilippis and Moore 2000; Galtier et al. 2009; Xavier et al. 2015). In most cases, these limitations can be overcome by sequencing additional markers or by sequencing complete mitochondrial or nuclear genomes, which provides greater statistical power (Morin et al. 2010; Emami‐Khoyi 2023). However, no focused comparison has been made using this approach to differentiate between *O. vulgaris* Type III and other closely related taxa.

In this study, we employed a combination of randomly sequenced intronic nuclear markers and the complete mitochondrial genomes of two *O. vulgaris* Type III specimens from South Africa and Amsterdam Island to assess the phylogenetic placement of this taxon within the species complex. The present results, which are based on substantially more molecular data than previous studies, support the hypothesis that *O. vulgaris* Type III is a genetically distinct taxon that requires a comprehensive taxonomic re‐evaluation.

## 2 Methods

### 2.1 Sample Collection, Genomic Library Preparation and Sequencing

Tissue samples were taken from two specimens of *Octopus vulgaris* Type III, one collected in False Bay, South Africa (34^o^13′S, 18^o^38′E) and the other from Amsterdam Island in the southern Indian Ocean (37^o^50′S, 77^o^33′E) (Figure 1).

DNA of high molecular weight was extracted from a small piece of muscle tissue using the Purification of Total DNA from Animal Tissues (Spin‐Column Protocol) of the QIAGEN DNeasy Blood & Tissue kit (Hilden, Germany). A genomic library was prepared from the extracted DNA using the NOVO kit (Novogene, Beijing, China). Briefly explaining, genomic DNA was first sheared into smaller fragments, and only a subset of fragments around~350 bp in length was selected for library preparation. The quality of the genomic library was assessed using a combination of Qubit (Thermo Fisher Scientific, Waltham, USA), qPCR, and the DNA NGS 3 K assay (PerkinElmer, Waltham, USA). The quality‐checked genomic library was then sequenced on a NovaSeq 6000 SP platform (Illumina, San Diego, USA) using the paired‐end 250 protocol.

### 2.2 Contig Assembly and Assignment of Nuclear Markers

The quality of the raw sequences was visually checked using FastQC v012 (https://www.bioinformatics.babraham.ac.uk/projects/fastqc/). The raw sequences were then assembled into longer contigs using the MEGAHIT v.1.2 assembler in default mode, and genomic features were annotated using the GALBA v1.0 pipeline (Stanke et al. 2006; Buchfink et al. 2015; Hoff and Stanke 2019; Bruna et al. 2023). GALBA uses nuclear protein sequences from a closely related species, in this case *O. sinensis* (NCBI annotation GCF_006345805.1), to predict protein‐coding genes. It is particularly suitable for genome annotation when no RNA or protein evidence from the studied species is available, as was the case in this study. The coordinates of annotated genomic features were extracted from the resulting GFF (Gene Feature Format) annotation file and converted into BED format using a custom‐made UNIX script. The corresponding sequences for each feature were then extracted using BEDtools v2.31 (Quinlan and Hall 2010), and all features annotated as introns were used for the next step.

### 2.3 Reciprocal BLAST and Phylogenomic Tree Reconstruction

Whole nuclear genomes from nine closely related species were downloaded from the NCBI genome database (Table S1). These included a single representative of all species for which such data were published in the NCBI database as of August 2025, in addition to the two published *O. vulgaris* ss genomes, both of which were generated from octopuses caught in the Mediterranean. A reciprocal BLAST search was then performed to identify intronic loci that are homologous in all species. To minimise the likelihood of identifying paralogous sequences as the best reciprocal matches, only a subset of unique reciprocal best hits with a minimum similarity of 90% across a minimum length of 800 bp was considered for downstream phylogenomic analysis. These homologous loci were then aligned using MAFFT v7 (Katoh et al. 2002) by selecting the ‘auto’ flag, which automatically selects an appropriate alignment algorithm according to the characteristics of the data. Any low‐quality segments in a particular alignment that could not be aligned reliably were then removed using ClipKit v2.4 (Steenwyk et al. 2020) in default mode.

Phylogenomic relationships among the selected taxa were assessed by first constructing an exploratory consensus Approximately‐Maximum‐Likelihood tree in FastTree2 v2.2 (Price et al. 2010) using the GTR substitution model (Tavaré 1986). The aim of this step was to assess whether genotyped nuclear loci have enough statistical power to distinguish between selected taxa. Then, Bayesian Inference (BI) in BEAST2 v2.7 (Bouckaert et al. 2019) and Maximum Likelihood (ML) in IQ Tree2 v2.4 (Minh et al. 2020) were used to investigate evolutionary relationships in more detail. In BEAST2, a multi‐locus species tree was reconstructed using the StarBeast2 pipeline (Heled and Drummond 2010). For the tree search step, the optimal nucleotide substitution model for each locus was predicted using the bModelTest model‐averaging method (Bouckaert and Drummond 2017), which simultaneously estimates the optimal tree topology and the best substitution model within a reverse‐jump MCMC framework. BEAST2 was run with 50 MCMC chains, each with 999 million iterations following 200 million burn‐in steps. Convergence of the chains in terms of Effective Sample Size (ESS) was checked using Tracer v1.7 (Rambaut et al. 2018) and the coda v0.19‐4 R package (Plummer et al. 2006). A maximum clade credibility tree, using median heights and a 30% burn‐in, was generated in TreeAnnotator v2.7 (Bouckaert et al. 2019). The multi‐locus species trees were visualised using DensiTree v3.0 (Bouckaert 2010).

In IQTree2, the optimal substitution model for each locus was identified using the Model Finder method (Minh et al. 2013), implemented in the software, and the most likely substitution model based on the estimated Bayesian Information Criterion BIC was used for the phylogenetic tree reconstruction step. Bootstrap support for each bifurcation in the resulting ML tree was assessed through 99,999 rapid bootstrap replicates. The consensus topology of the BI and ML phylogenomic trees was visualised in Figtree v1.4.3 (Rambaut 2016).

### 2.4 Mitogenome Assembly and Annotation

Two mitochondrial genomes were assembled *de novo* using the GetOrganelle v.1.7 pipeline (Jin et al. 2020). The assembly parameters were set to their defaults, except for the k‐mer values, which were set for a combination of the following: 21, 45, 65, 85 and 105. The assembled mitogenomes were then submitted to the MITOS Web Server (Bernt et al. 2013; Donath et al. 2019) for the annotation of protein‐coding genes, tRNAs, and rRNAs. The predicted gene boundaries and the putative control region were manually adjusted in MEGA11 (Tamura et al. 2021) using the mitogenome of the previously published *O. sinensis* (NC_052881.1) from China (Li et al. 2021) as a template reference. The nucleotide composition of the complete mitogenome was calculated manually using the formulas AT‐skew = (A‐T)/(A + T) and GC‐skew = (G‐C)/(G + C) (Perna and Kocher 1995). Additionally, relative synonymous codon usage was estimated using the Ezmito tool (Cucini et al. 2021). The structures of 22 tRNAs and two rRNAs were predicted using MiTFi (Jühling et al. 2012) and the RNA‐fold server (Hofacker 2003, http://rna.tbi.univie.ac.at/cgi‐bin/RNAWebSuite/RNAfold.cgi), respectively, and visualised using the Forna webserver (http://rna.tbi.univie.ac.at/forna/). The annotated mitogenomes were visualised in Chloroplot (Zheng et al. 2020). The arrangement of mitogenomic features in *O. vulgaris* Type III was compared to those of other members of this genus by conducting a gene order analysis in CREx (Bernt et al. 2007).

### 2.5 Phylogenetic Analysis of Mitogenomes

The phylogenetic placement of *O. vulgaris* Type III mitogenomes among the mitogenomes of other *Octopus* species was assessed using Bayesian inference (BI) and maximum likelihood (ML) methods. First, all mitogenomes from species belonging to the genus *Octopus* (*n* = 57) were retrieved from the NCBI nucleotide database. The dataset currently includes the complete mitogenomes of 10 species within this genus, including mitogenomes of *O. vulgaris* ss (OR353425.1) and two mitogenomes of *O. sinensis* (NC_052881.1 and NC_006353.1). The sequence with accession number NC_006353.1 is listed on NCBI as *O. vulgaris* , but Fee et al. (2024) demonstrated that this mitogenome is incorrectly labelled and provided evidence that it originates from an *O. sinensis* specimen. The Ezsplit tool was used to extract the sequences of all 13 protein‐coding genes (PCG) from the database, each of which was aligned separately using the MAFFT sequence alignment tool using the same algorithm that was used for nuclear sequences (Katoh et al. 2018).

A BI phylogenetic tree was reconstructed using BEAST2 v2.5 (Bouckaert et al. 2019). For each gene, the best substitution model was predicted using bModelTest and BEAST2 was run with 20 MCMC chains, each with 500 million iterations following an initial burn‐in of 100 million iterations. A maximum clade credibility tree using median heights and a 30% burn‐in was then constructed in TreeAnnotator. The ML phylogenetic tree was constructed using IQ‐TREE v2.4, selecting the most likely substitution model as explained in the previous section. Both BI and ML trees were visualised in Figtree.

### 2.6 Species Delimitation Analysis

As a molecular investigation into whether the branch leading to *O. vulgaris* Type III could represent an undescribed species within the *O. vulgaris* complex, an integrative approach was used that combined the bPTP species delimitation pipeline (Zhang et al. 2013) and the Assemble Species by Automatic Partitioning (ASAP) stand‐alone software (Puillandre et al. 2021). The bPTP method uses an ultrametric phylogenetic tree to identify putative species boundaries, whereas ASAP utilises pairwise genetic distance among sequences and performs a series of hierarchical clustering to predict the most probable genetic partitions. The genetic distance thresholds for species partitioning in this method are automatically calculated, making the results independent from an arbitrary user‐defined threshold.

## 3 Results

### 3.1 Contig Assembly

The Illumina sequencing runs produced 12,080,692 and 9,975,641 paired‐end sequences for the South African and Amsterdam Island specimens, respectively, with an average Phred quality score of 36. The MEGAHIT pipeline assembled sequences generated from South African specimens into 2,048,145 contigs ranging in size from 200 bp to 16,171 bp with an average length of 704.7 bp. In the specimen collected from Amsterdam Island, 1,795,109 contigs, ranging in size from 200 to 15,805 bp, with an average length of 663.4 bp, were assembled.

### 3.2 Reciprocal BLAST and Assignment of Nuclear Markers

Annotating assembled contigs based on the protein‐coding sequences from the closely related *O. sinensis* resulted in the identification of 49,621 putatively intronic sequences with an average length of 1873.9 bp from the South African specimen, and 21,314 sequences with an average length of 1893.4 bp from the Amsterdam Islands specimen. After performing a reciprocal blast between the extracted sequences from the two studied species and those of closely related octopuses, a subset of 338 homologous regions, ranging in size from 776 bp to 8498 bp with an average size of 2030 bp, was selected for phylogenomic analysis.

### 3.3 Phylogenomic Tree Reconstruction

**FIGURE 2:**
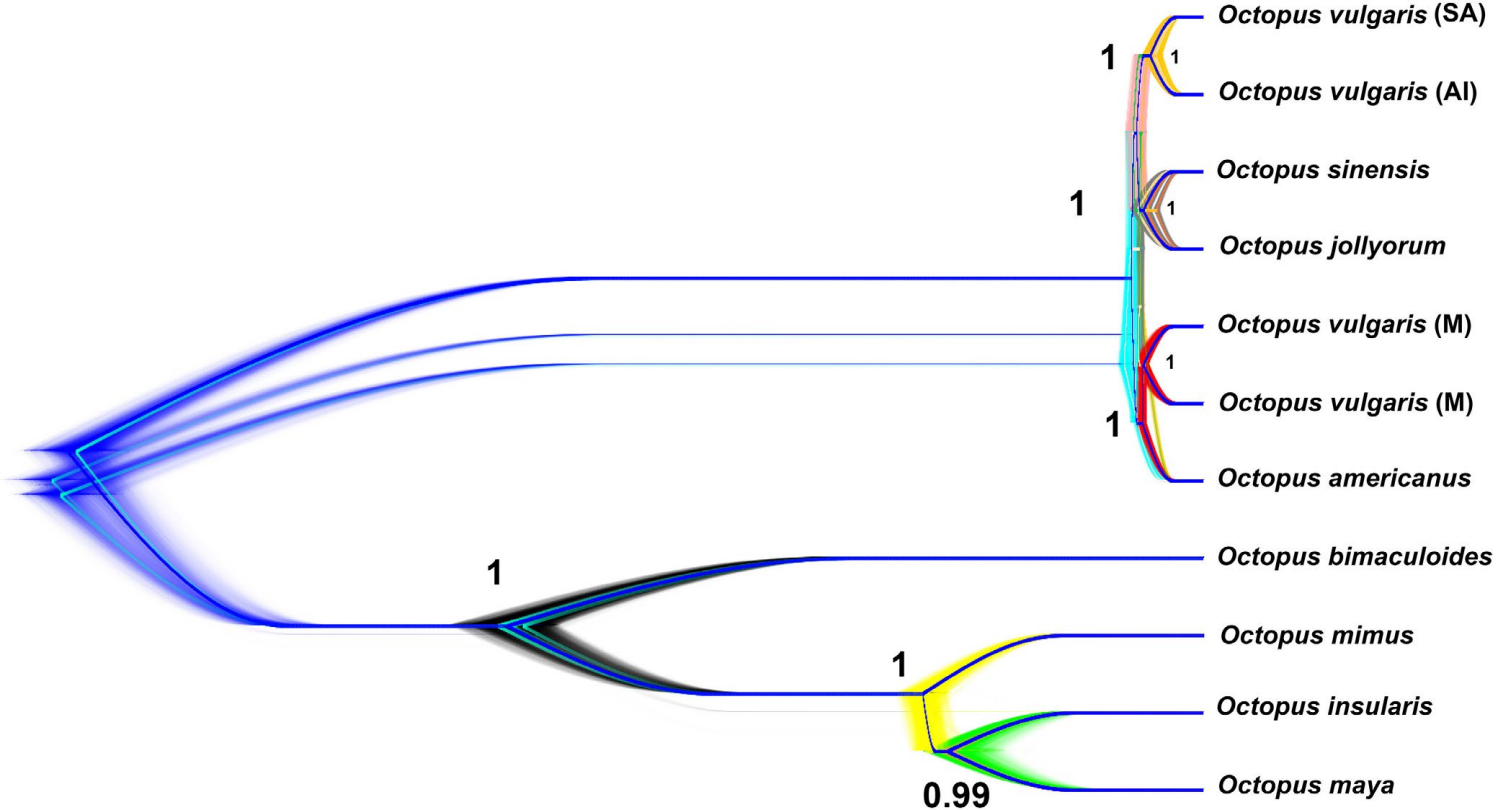
DensiTree visualisation of 100,000 StarBEAST2 species trees reconstructed using 338 randomly sequenced nuclear markers. Clades have been shown using different colours. The acronyms SA, AI and M indicate specimens collected in South Africa, Amsterdam Island and the Mediterranean Sea, respectively.

The topologies of the consensus Approximately‐Maximum‐Likelihood tree generated by FastTree2, the Bayesian species tree inferred using BEAST2, and the Maximum‐Likelihood tree produced by IQ‐TREE 2 were congruent, with strong statistical support observed across all trees in terms of Posterior probability, tree topologies, and bootstrap supports (Figure 2 and Figure S1).

The lineage comprising specimens from South Africa and Amsterdam Island constituted a distinct clade, which was sister to a clade containing *O. jollyorum* from the South Pacific and *O. sinensis* from East Asia. These two clades formed a monophyletic group with *O. vulgaris* ss from the Mediterranean Sea.

### 3.4 Mitogenome Assembly and Annotation

**FIGURE 3:**
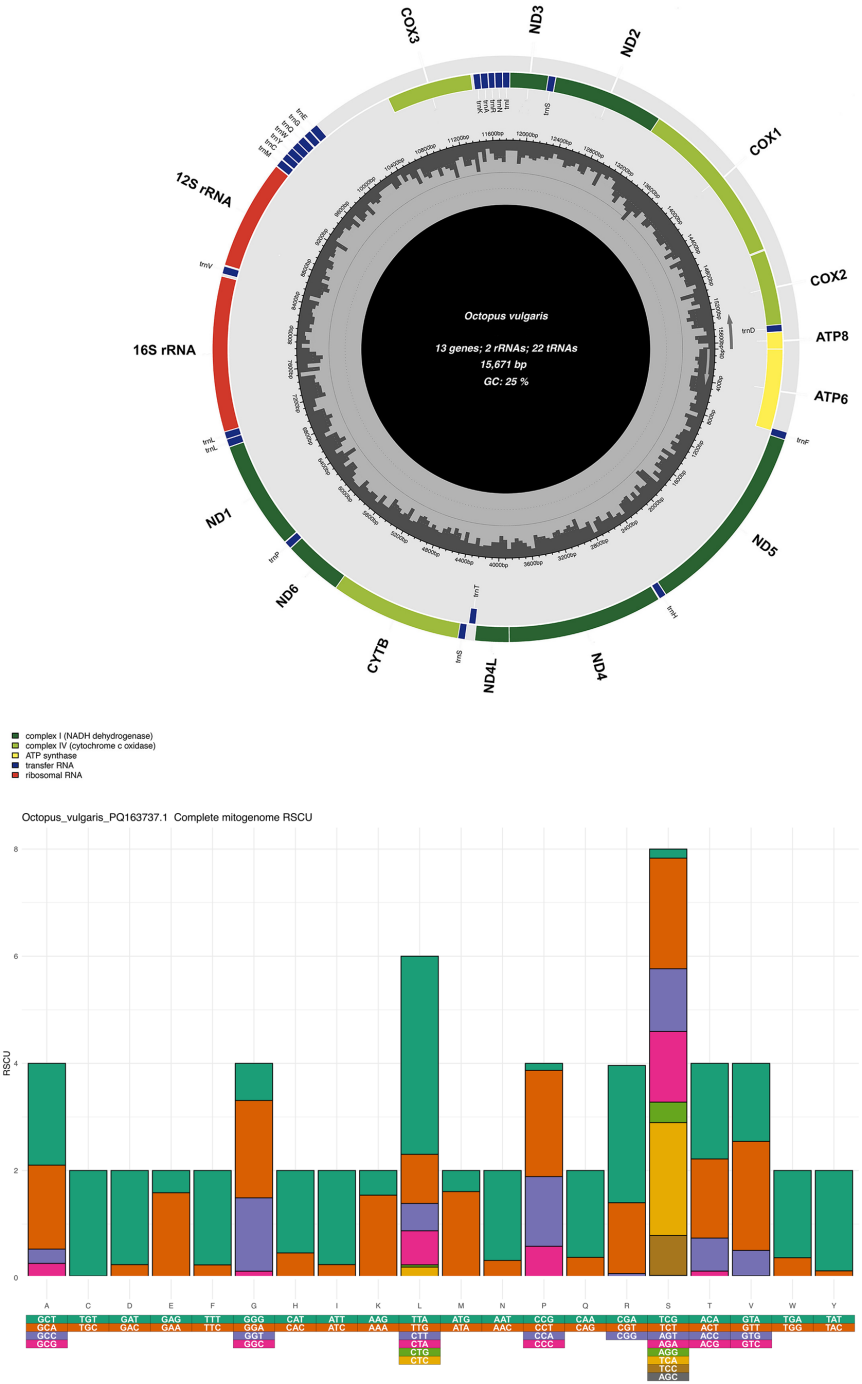
Graphic representation of the South African *Octopus vulgaris* Type III mitogenome, indicating the location of tRNAs (blue), rRNAs (red), and protein‐coding genes (green). Grey bars in the inner circle represent GC content (Top). Relative Synonymous Codon Usage for the same species (Bottom).

The GetOrganelle pipeline produced two circular contigs: a 15,671 bp contig with a GC content of 25% for the South African specimen (Figure 3a) and a 15,663 bp contig with a GC content of 25.1% for the specimen from Amsterdam Island.

Since the two assembled mitogenomes were 99.8% identical, only the South African specimen is discussed here in detail as representative of the Type III lineage. A graphical representation of the Amsterdam Island specimen is included in the Figure S2. The nucleotide frequencies of the South African specimen are A = 41%, C = 17.4%, G = 7.6% and T = 34%. The mitogenome has a positive AT skew (0.093) and a negative GC skew (−0.392). The MITOS annotation pipeline identified 13 PCGs, 22 transfer RNAs (tRNA) and two ribosomal RNAs (rRNA), consistent with those reported from other *Octopus* species. The plus strand contained seven PCGs (*atp6*, *atp8*, *cox1*, *cox2*, *cox3*, *nad2*, *nad3*) and eight tRNAs, while the minus strand contained 6 PCGs (*cytb*, *nad1*, *nad4*, *nad4l*, *nad5*, *nad6*), 14 tRNAs and both rRNAs. All PCGs start with ATG, except for nad4 and nad4l, which both start with ATA. The putative control region was approximately 700 bp in length and located between *cox3* and tRNA E (UUC), with a GC content of 17.9%. The annotated control regions were 99.6% identical between the two specimens. Several shorter intergenic sequences (*n* = 21), ranging in length from 1 to 81 bp, were also identified. The synonymous relative codon usage in the assembled mitogenomes was comparable to that reported from other, closely related octopus species (Figure 3b). The predicted structures of annotated tRNAs and rRNAs were the typical cloverleaf and multi‐loop structures, respectively (Figure S3). No gene order rearrangement compared to other members of the *O. vulgaris* complex was observed.

The NCBI BLAST results showed that the two *Octopus vulgaris* Type III sequences from South Africa were most similar to *O. vulgaris* ss, with 98.76% and 98.68% identity (OR353425.1 and OY720585.1, respectively). *Octopus sinensis* shared 96.3% and 96.2% identity (NC052881.1 and NC006353.1), while the similarity of the representative assembled mitogenome with *O. mimus* (NC044093.1) and *O. bimaculoides* (NC029723.1) was 85.0% and 85.2%, respectively.

**FIGURE 4:**
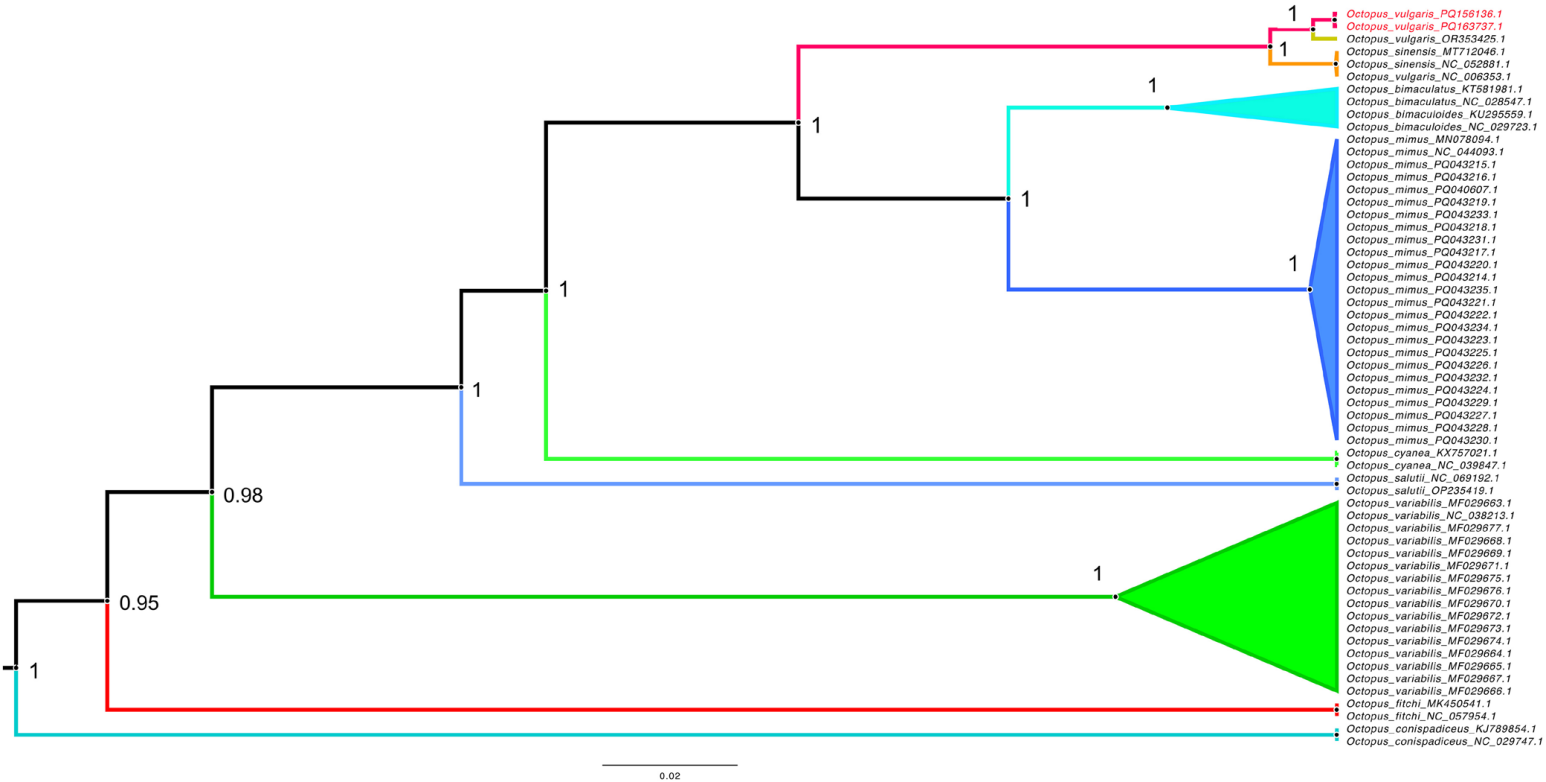
A maximum clade credibility consensus Bayesian phylogenetic tree reconstructed using 13 protein‐coding genes. Bayesian posterior probabilities are shown at each node. The lineage leading to the two *Octopus vulgaris* Type III has been highlighted in red.

The observed pattern was reflected in both the BI (Figure 4) and ML (Figure S4 and Table S2) phylogenetic reconstructions, where the *O. vulgaris* Type III mitogenomes formed a clade with a sister taxon relationship to *O. vulgaris* ss. In both cases, the topology of the phylogenetic trees was supported by high Bayesian posterior probabilities and fast bootstrap values.

**FIGURE 5:**
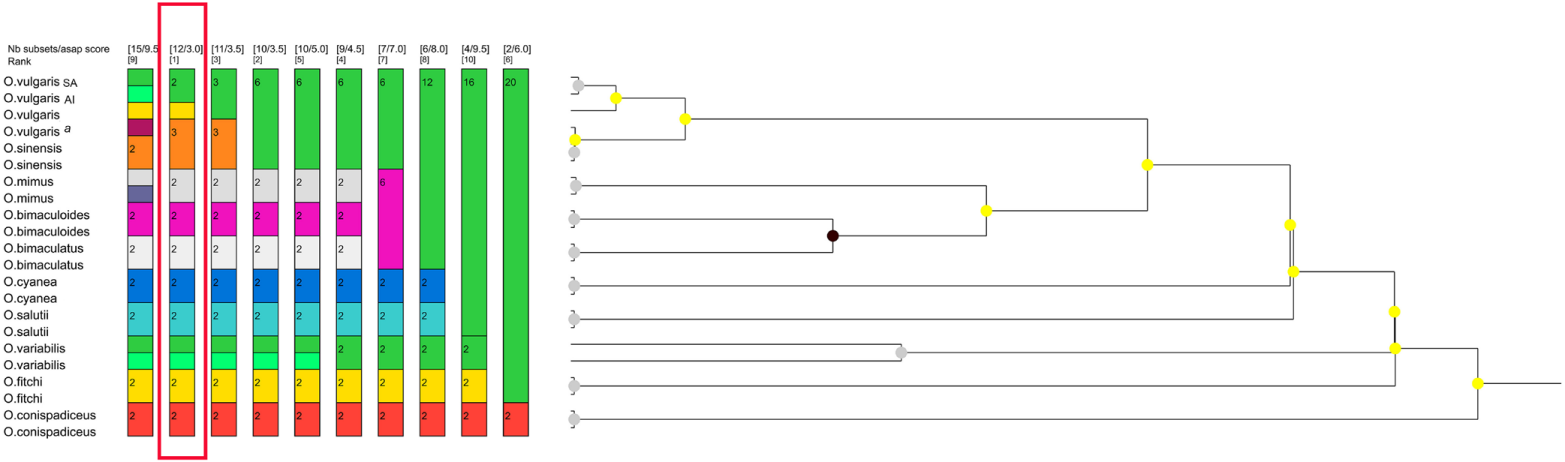
Assemble Species by Automatic Partitioning (ASAP) analyses of different species in the octopus genus based on 13 mitochondrial protein‐coding genes. The columns on the left illustrate putative groups of species. Groups of sequences representing a single species are indicated by colours, with numbers indicating how many sequences were grouped into a putative species Numbers above each column indicate the ASAP‐score coefficient (the lower value), the putative number of species (the upper right value), and the ranking of the splitting lower value pattern compared to all alternative splitting scenarios. The red rectangle indicates the highest‐ranked assignment of species. This scenario clearly illustrates the distinction between the *O. vulgaris* Type III and other members of the *O. vulgaris* complex.

Bayesian species delimitation analysis of the reconstructed ultrametric tree in BEAST2 showed that the lineage leading to *O. vulgaris* Type III is genetically distinct and could represent a new species with 0.73 posterior support. A further split of *O. vulgaris* Type III into distinct South African and Amsterdam Island lineages was not supported. Similarly, the results of the ASAP distance‐based species delimitation rank the speciation scenario with a split between *O. vulgaris* ss and *O. vulgaris* Type III as the most likely scenario among all alternative speciation scenarios (*p* = 0.03, Figure 5).

## 4 Discussion

The identification of cryptic species within wild populations holds significant implications for the conservation of marine resources (Knowlton 1993). Molecular data provide useful information for investigating population structure, gene flow, and phylogenetic relationships (Moritz et al. 1987), which includes the identification and delineation of closely related species (Morin et al. 2010). In this study, we employed a combination of 338 randomly sequenced intronic nuclear markers in addition to two complete mitochondrial genomes from two specimens of *Octopus vulgaris* Type III from South Africa and Amsterdam Island to assess their taxonomic placement relative to closely related octopus species.

Numerous studies have highlighted that elucidating phylogenetic relationships among closely related species using a limited number of genetic markers can be inconclusive and is influenced by taxon‐specific genetic variability (DeFilippis and Moore 2000; Galtier et al. 2009; Morin et al. 2010). For example, while phylogenies based on *cox1*, *cox3*, or *16S rRNA* could not provide conclusive species‐level resolution to distinguish between *O. vulgaris* ss and Type III due to the low genetic variation in the selected markers (Warnke et al. 2004; Oosthuizen et al. 2004), Amor et al. 2014 used a different combination of mitochondrial markers—including *12S rRNA*, *16S rRNA*, c*ox1*, *cox3*, and *cytb*—to prove allopatric speciation among Australasian octopuses. Similarly, variation within the control region and the *cox1* gene of *O. vulgaris* populations from the northeastern Atlantic was sufficient to distinguish two distinct haplotypes, indicating significant population structure among populations (Quinteiro et al. 2020).

The concurrent analysis of nuclear loci alongside complete mitochondrial genomes in this study demonstrated a clear genetic distinction between *O. vulgaris* Type III and *O. vulgaris* ss. However, the phylogenetic tree topologies generated from nuclear and mitochondrial markers displayed discordance. In the phylogenetic tree reconstructed using nuclear markers, the southern hemisphere lineage described in this study was identified as a sister taxon to a lineage comprising *O*. *jollyorum* from the South Pacific and *O. sinensis* from East Asia, with only the most recent common ancestor of these two lineages forming a sister group to the lineage leading to *O. vulgaris* from the Mediterranean Sea. Conversely, in the tree derived from 13 mitochondrial protein‐coding genes, this southern clade was recovered as a sister taxon to *O. vulgaris* ss from the Mediterranean Sea, and the divergence of this clade from *O. sinensis* was inferred to be more ancient.

Amor et al. (2019) were already aware of the discrepancies between the nuclear and mitochondrial markers found in this study and suggested that this could be a result of population isolation followed by secondary contact and hybridisation. *Octopus vulgaris* ss and *O. vulgaris* Type III have allopatric distributions in the northern and southern hemispheres, respectively. Although octopus larvae are planktonic and have large dispersal capabilities, there is a distribution gap in this species complex across tropical equatorial West Africa. In addition, the Lüderitz upwelling cell in southern Namibia potentially represents a cold‐water barrier (Lett et al. 2007), marking the north‐western edge of the distribution range of *O. vulgaris* Type III. Due to this barrier and the large geographic separation between species, contemporary gene flow is unlikely to occur, although it may have been possible in the past.

Evidence of trans‐oceanic dispersal has already been suggested for another member of this genus, *O. insularis* . In a passive dispersal model, Lima et al. (2023) showed that the paralarvae of this American species traversed the Atlantic Ocean and established new populations on African coasts. However, such events are likely to have occurred during glacial phases during the Miocene–Pliocene epochs, when sea levels were lower (Lima et al. 2023). Similar dispersal events may have occurred during the evolutionary history of the southern lineage, the full scope of which remains unknown.

As an alternative explanation, recent unintentional introductions through ships' ballast water may have happened (Williams et al. 1988; Bello et al. 2020). This process would lead to the introgression of nuclear or mitochondrial genetic material into the genomes of *O. vulgaris* Type III. The nature and magnitude of such admixture remain undetermined, necessitating a comprehensive genome‐wide analysis of variation within *O. vulgaris* Type III populations throughout their entire distribution range using much larger sample sizes than were used in this study.

The complete mitogenome of the *Octopus vulgaris* group III was 98.8% similar to that of *O. vulgaris* ss and 96.6% similar to *O. sinensis* . Similarly, the Kimura two‐parameter distance (K2P, Kimura 1980) of the *cox1* gene of *O. vulgaris* Type III differed by 1% from *O. vulgaris* ss and 2.6% from *O. sinensis* . When summarising *cox1* variation, Undheim et al. (2010) found that the intraspecific difference within *O. vulgaris* ranged from 0% to 1.3%. While genetic divergence of > 2% is often considered to corroborate accepted species boundaries (Roux et al. 2016). Many species have been delineated on much lower levels of genetic divergence, for example, as little as 1%–2% in the octopod genus *Pareledone* (Allcock et al. 2007), large white‐headed gulls (Crochet et al. 2003) and killer whales (Morin et al. 2010), and even as low as 0.075% in a pair of fairywren species (Roux et al. 2016).

Small genetic distances between mitochondrial haplotypes similar to those observed between the southern lineage and *O. vulgaris* ss may also arise due to hybridisation events or the recent divergence of lineages (Hebert et al. 2004). Amor et al. (2019) reported that the *O. vulgaris* species complex emerged within the past 2.5 million years, with the divergence between the *O. vulgaris* ss and *O. vulgaris* Type III lineages estimated to have occurred approximately 1.0–1.5 million years ago. In the absence of precise estimates regarding the intraspecific mitogenomic variation of *O. vulgaris* Type III across its distributional range, relying exclusively on genetic distance to ascertain the taxonomic status of this southern lineage is problematic. Investigating this topic offers a promising direction for future studies.

The molecular data presented in this study support the idea that *O. vulgaris* Type III constitutes a distinct genetic lineage within the *O. vulgaris* complex. However, a thorough integrative phylogenetic analysis incorporating additional morphological and ecological evidence is essential to conclusively re‐evaluate and resolve the current taxonomic status of this undescribed southern hemisphere lineage.

## 5 Conclusion

Despite multiple recent studies investigating the *Octopus vulgaris* species complex, the identity of the southern hemisphere lineage that occurs in the southeastern Atlantic and in the southern Indian Ocean remained unresolved. We conducted the first phylogenomic analysis demonstrating the genome‐wide distinctiveness of the South African and Amsterdam Island lineages in comparison to their closest relatives, *O. vulgaris* sensu ss from the Mediterranean and *O. sinensis* from East Asia. These results contribute to recent advances aimed at resolving the systematics of the *O. vulgaris* complex.

## Author Contributions

**Arsalan Emami‐Khoyi:** conceptualization (equal), data curation (equal), formal analysis (equal), software (equal), supervision (equal), validation (equal), visualization (supporting), writing – review and editing (supporting). **Gareth N. Fee:** conceptualization (equal), data curation (equal), formal analysis (equal), methodology (equal), project administration (equal), software (equal), validation (equal), visualization (equal), writing – original draft (equal). **Jannes Landschoff:** investigation (supporting), project administration (supporting), resources (supporting), supervision (supporting), validation (supporting), writing – review and editing (supporting). **Michael D. Amor:** methodology (supporting), validation (supporting), writing – review and editing (supporting). **Charles Griffiths:** resources (supporting), validation (supporting), writing – review and editing (supporting). **Yves Cherel:** resources (equal), writing – review and editing (supporting). **Peter R. Teske:** conceptualization (equal), data curation (equal), formal analysis (equal), funding acquisition (equal), methodology (equal), project administration (equal), resources (equal), supervision (equal), validation (equal), writing – review and editing (supporting).

## Conflicts of Interest

The authors declare no conflicts of interest.

## Supporting information


**Data S1:** ece373235‐sup‐0001‐supinfo.docx.

## Data Availability

The South African specimen has been donated to the Iziko South African Museum in Cape Town (voucher number: Oct_TypeIII_FB2022_01). The two annotated mitogenomes have been submitted to the NCBI nucleotide database under accession numbers PQ163737 (South Africa) and PQ156136 (Amsterdam Island). The raw sequences for this study are available at NCBI databases under BioProject PRJNA1145185 and SRA accession SRR30158481.
