# Supplementary material for: Genomic Divergence Between Octopus vulgaris and Its Undescribed Sister Species From the South Atlantic and Indian Ocean
Source: Ecol Evol. 2026 Mar 16;16(3):e73235. doi: 10.1002/ece3.73235 (PMC13093632; doi:10.1002/ece3.73235)
Supplement: Supplementary file 1 — Data S1: ece373235‐sup‐0001‐supinfo.docx. [file ECE3-16-e73235-s001.docx]

**Table S1**: NCBI Accession number of assemblies downloaded for phylogenomic analysis in this study.

| **Assembly Accession** | **Assembly Name** | **Organism Name** | **Assembly Level** | **Number of Scaffolds** |
| --- | --- | --- | --- | --- |
| GCA_001194135.2 | ASM119413v2 | *Octopus bimaculoides* | Chromosome | 145326 |
| GCA_006345805.1 | ASM634580v1 | *Octopus sinensis* | Chromosome | 13515 |
| GCA_027122535.1 | ASM2712253v1 | *Octopus maya* | Scaffold | 198627 |
| GCA_027568975.1 | ASM2756897v1 | *Octopus mimus* | Scaffold | 213283 |
| GCA_029874955.1 | RU_OctIns_1.0 | *Octopus insularis* | Contig | 1302592 |
| GCA_034509045.1 | ASM3450904v1 | *Octopus jollyorum* | Scaffold | 94659 |
| GCA_027568995.1 | ASM2756899v1 | *Octopus americanus* | Scaffold | 108890 |
| GCA_951406725.2 | xcOctVulg1.2 | *Octopus vulgaris* | Chromosome | 226 |
| GCA_003957725.1 | ASM395772v1 | *Octopus vulgaris* | Scaffold | 77681 |

**Table S2**: The best nucleotide substitution models predicted by IQtree that were used to reconstruct a Maximum-Likelihood phylogenetic tree from 13 mitochondrial protein-coding genes.

| **Feature** | **The best substitution model based on BIC** |
| --- | --- |
| Concatenated genes | TVM+F+I+G4 |
| *atp6* | TIM2+F+G4 |
| *atp8* | HKY+F+I |
| *cox1* | TIM2+F+I+G4 |
| *cox2* | TIM2+F+I+G4 |
| *cox3* | TIM2+F+I+G4 |
| *Cytb* | TIM3+F+I+G4 |
| *nad1* | TIM+F+I+G4 |
| *nad2* | TIM3+F+I+G4 |
| *nad3* | TIM2+F+G4 |
| *nad4* | TIM+F+I+G4 |
| *nad4l* | HKY+F+G4 |
| *nad5* | TIM+F+I+G4 |
| *nad6* | HKY+F+I+G4 |

**Figure S1** : Maximum likelihood phylogenomic tree based on 338 randomly sequenced nuclear markers. Clade of Octopus vulgaris Type III is highlighted in red. The acronyms ZA, AI and M indicate specimens collected in South Africa, Amsterdam Island, respectively. The value on each node represents fast bootstrap support for that split based on 99,999 rapid bootstraps.


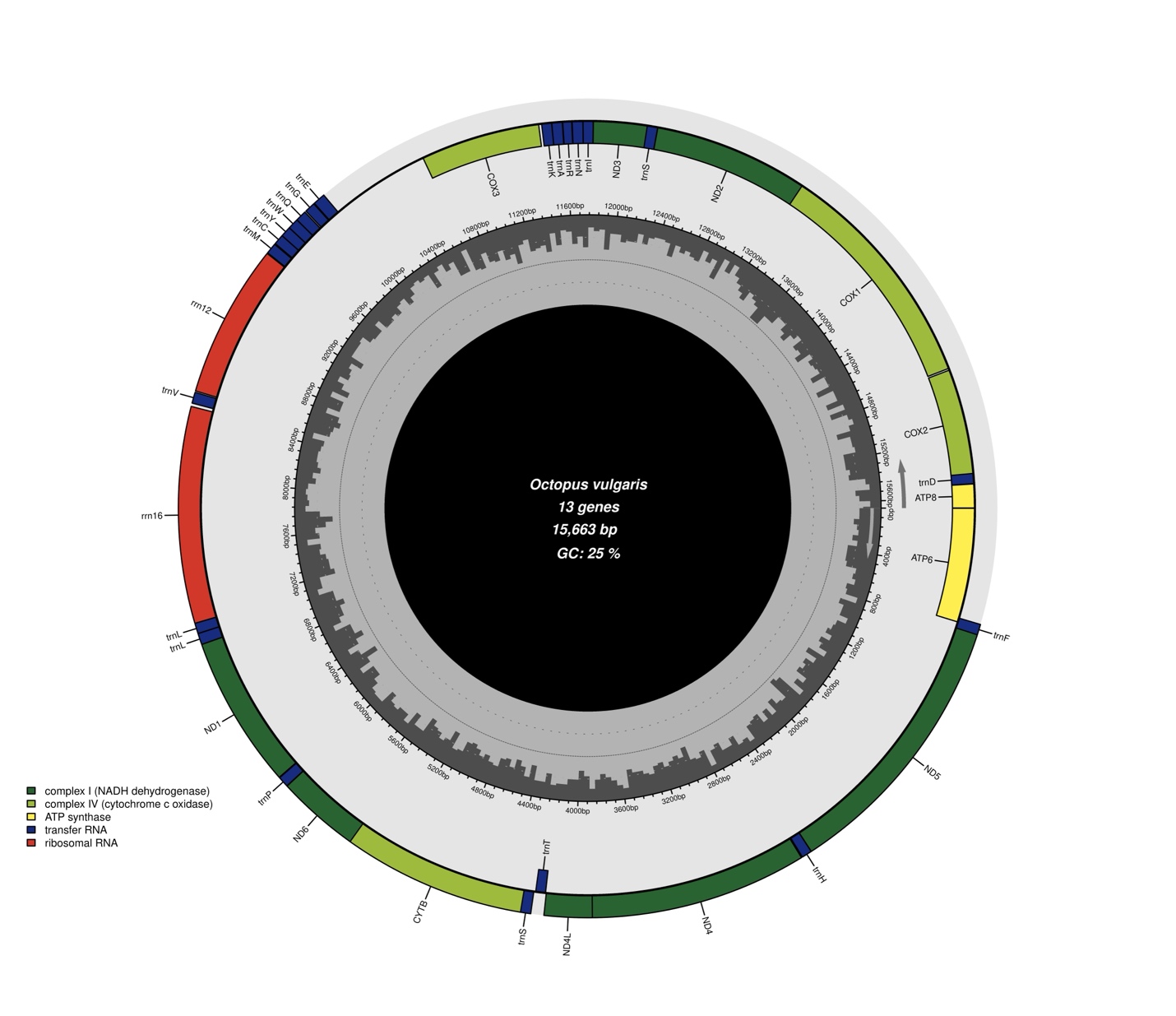


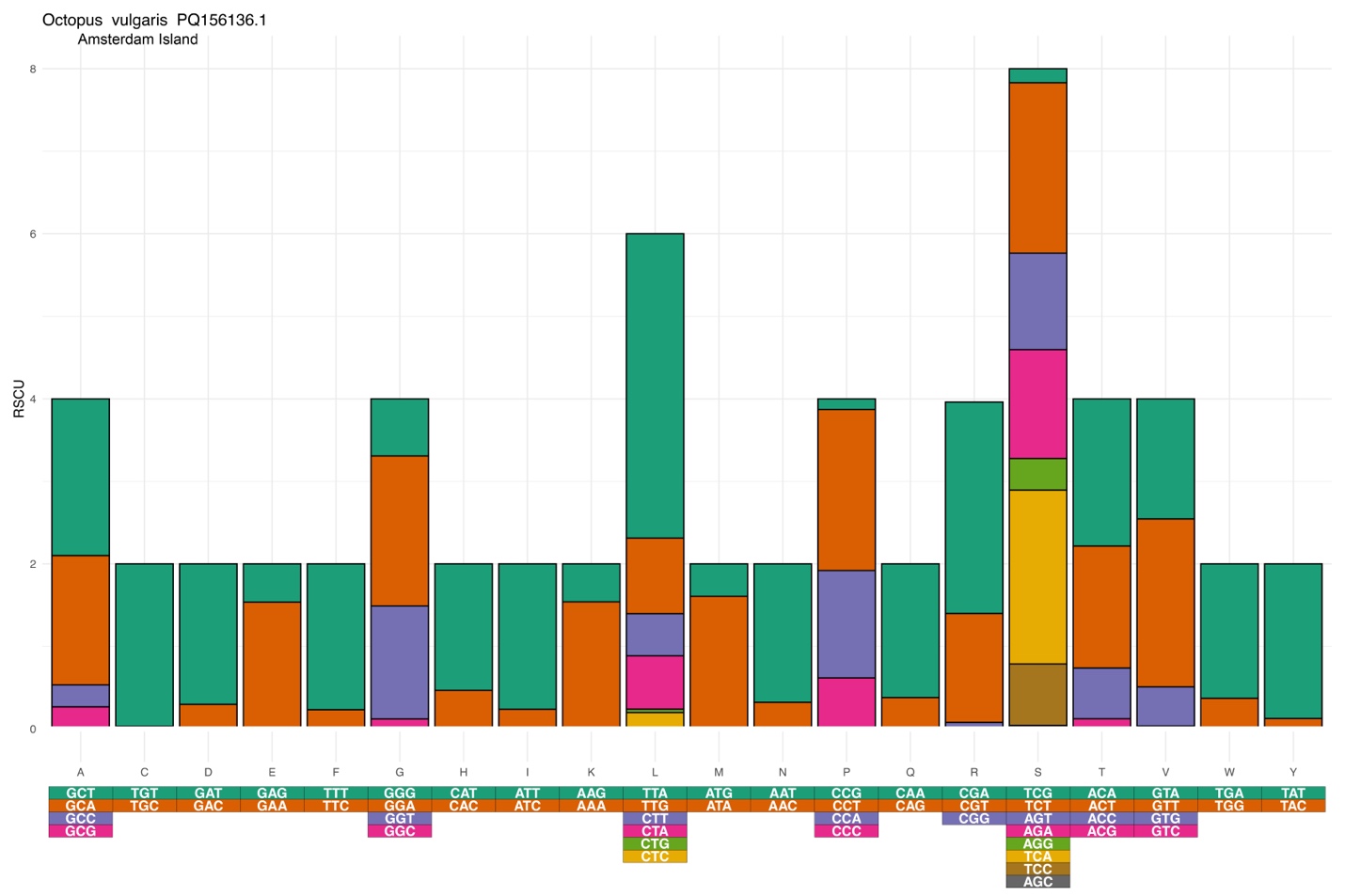


**Figure S2:** Graphic representation of the South African Octopus vulgaris Type III mitogenome, indicating the location of tRNAs (blue), rRNAs (red), and protein-coding genes (green). Grey bars in the inner circle represent GC content (Top). Relative Synonymous Codon usage in the O.vulgaris Type III mitogenome(bottom).


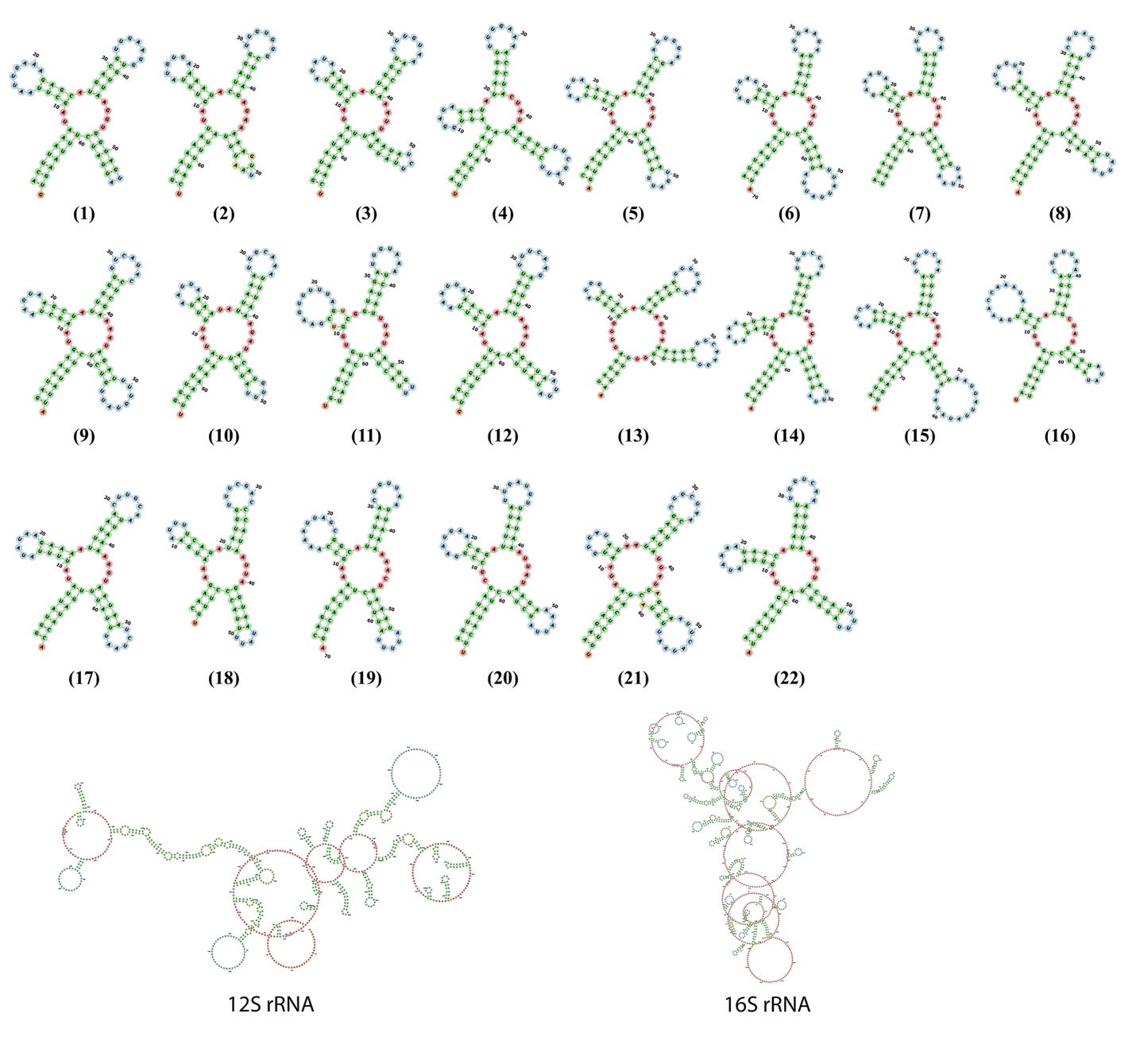


**Figure S3:** Predicted structure of 22 tRNAs and two rRNAs in Octopus vulgaris Type III mitogenome. Numbers 1 to 22 corresponds to GAA(F), GUG(H), UGU(T), UGA(S2), UGG(P), UAA(L2), UAG(L1), UAC(V), CAU(M), GCA(C), GUA(Y), UCA(W), UUG(Q), UCC(G), UUC(E), UUU(K), UGC(A), UCG(R), GUU(N), GAU(I), GCU(S1), and GUC(D) tRNA , respectively.

**Figure S4**: Maximum likelihood phylogenetic tree reconstructed using 13 protein-coding genes. The lineage leading to O. vulgaris Type III has been highlighted in red. The value on each node shows the fast bootstrap support for the split.
